# Supplementary material for: Potent neutralization of Marburg virus by a vaccine-elicited antibody
Source: Nature. 2025 Nov 12;650(8101):459–69. doi: 10.1038/s41586-025-09868-1 (PMC12893919; doi:10.1038/s41586-025-09868-1)
Supplement: Supplementary file 1 — Reporting Summary [file 41586_2025_9868_MOESM1_ESM.pdf]

nature portfolio

Corresponding author(s): David Veesler; Fabio Benigni

Last updated by author(s): 09/30/2025

Reporting Summary

Nature Portfolio wishes to improve the reproducibility of the work that we publish. This form provides structure for consistency and transparency in reporting. For further information on Nature Portfolio policies, see our Editorial Policies and the Editorial Policy Checklist.

Statistics

For all statistical analyses, confirm that the following items are present in the figure legend, table legend, main text, or Methods section.

n/a	Confirmed	
☐	☒	The exact sample size (*n*) for each experimental group/condition, given as a discrete number and unit of measurement
☐	☒	A statement on whether measurements were taken from distinct samples or whether the same sample was measured repeatedly
☐	☒	The statistical test(s) used AND whether they are one- or two-sided<br>*Only common tests should be described solely by name; describe more complex techniques in the Methods section.*
☐	☒	A description of all covariates tested
☐	☒	A description of any assumptions or corrections, such as tests of normality and adjustment for multiple comparisons
☐	☒	A full description of the statistical parameters including central tendency (e.g. means) or other basic estimates (e.g. regression coefficient) AND variation (e.g. standard deviation) or associated estimates of uncertainty (e.g. confidence intervals)
☐	☒	For null hypothesis testing, the test statistic (e.g. *F*, *t*, *r*) with confidence intervals, effect sizes, degrees of freedom and *P* value noted<br>*Give P values as exact values whenever suitable.*
☒	☐	For Bayesian analysis, information on the choice of priors and Markov chain Monte Carlo settings
☒	☐	For hierarchical and complex designs, identification of the appropriate level for tests and full reporting of outcomes
☒	☐	Estimates of effect sizes (e.g. Cohen's *d*, Pearson's *r*), indicating how they were calculated

Our web collection on statistics for biologists contains articles on many of the points above.

Software and code

Policy information about availability of computer code

Data collection	Leginon; QuantStudio Design and Analysis Desktop Software; FACS DIVA (Version 9.0)
Data analysis	GraphPad Prism 10; Octet Data Analysis HT software v12.0; WARP; cryoSPARC; Relion; UCSF ChimeraX; Coot; AlphaFold2; Rosetta; ISOLDE; Phenix; QuantStudio Design and Analysis Desktop Software; Trimmomatic v0.39; Geneious Prime; FlowJo (version 10.8.0)

For manuscripts utilizing custom algorithms or software that are central to the research but not yet described in published literature, software must be made available to editors and reviewers. We strongly encourage code deposition in a community repository (e.g. GitHub). See the Nature Portfolio guidelines for submitting code & software for further information.

Data

Policy information about availability of data

All manuscripts must include a data availability statement. This statement should provide the following information, where applicable:

- Accession codes, unique identifiers, or web links for publicly available datasets
- A description of any restrictions on data availability
- For clinical datasets or third party data, please ensure that the statement adheres to our policy

The cryo-EM maps and atomic coordinates were deposited to the Electron Microscopy Data Bank (EMDB) and the PDB with accession numbers 9NJL and EMD-49486. Sequencing reads are available under NCBI BioProject PRJNA1336301.

Research involving human participants, their data, or biological material

Policy information about studies with human participants or human data. See also policy information about sex, gender (identity/presentation), and sexual orientation and race, ethnicity and racism.

Reporting on sex and gender	N/A
Reporting on race, ethnicity, or other socially relevant groupings	N/A
Population characteristics	N/A
Recruitment	N/A
Ethics oversight	N/A

Note that full information on the approval of the study protocol must also be provided in the manuscript.

Field-specific reporting

Please select the one below that is the best fit for your research. If you are not sure, read the appropriate sections before making your selection.

☒ Life sciences ☐ Behavioural & social sciences ☐ Ecological, evolutionary & environmental sciences

For a reference copy of the document with all sections, see nature.com/documents/nr-reporting-summary-flat.pdf

Life sciences study design

All studies must disclose on these points even when the disclosure is negative.

Sample size	No sample size calculation was performed to design the study. The number of mice used for antibody discovery and immunogenicity study and the number of Guinea pigs used for the challenge study were based on previous experience with the model.
Data exclusions	No data were excluded.
Replication	Experimental assays were performed at least in two independent biological replicates. Each replicate was performed with 1 to 6 technical replicates. All attempts at replication were successful.
Randomization	For the immunogenicity study, mice were randomly assigned to groups ensuring the groups had an equal number of females and males. For the challenge study, Guinea pigs were randomly assigned to groups. For the antibody discovery study, no randomization was performed as all antigen-positive memory B cells were evaluated.
Blinding	Blinding was not performed as this study is not a case control study.

Reporting for specific materials, systems and methods

We require information from authors about some types of materials, experimental systems and methods used in many studies. Here, indicate whether each material, system or method listed is relevant to your study. If you are not sure if a list item applies to your research, read the appropriate section before selecting a response.

Materials & experimental systems

n/a	Involved in the study	
☐	☒	Antibodies
☐	☒	Eukaryotic cell lines
☒	☐	Palaeontology and archaeology
☐	☒	Animals and other organisms
☒	☐	Clinical data
☒	☐	Dual use research of concern
☒	☐	Plants

Methods

n/a	Involved in the study	
☒	☐	ChIP-seq
☐	☒	Flow cytometry
☒	☐	MRI-based neuroimaging

Antibodies

Antibodies used	MARV4 (generated in-house); MARV7 (generated in-house); MARV11 (generated in-house); MARV12 (generated in-house); MARV14

Antibodies used	(generated in-house); MARV16 (generated in-house); MARV18 (generated in-house); MARV20 (generated in-house); MARV21 (generated in-house); MARV23 (generated in-house); MR78 (PDB: 5UQ, generated in-house); MR191 (PDB: 6BP2, generated in-house); EBOV-515 (PBD: 7KF9, generated in-house); Goat anti-human IgG Fc HRP (Thermo Fisher Scientific, catalog #A18823, 1:5,000 dilution);PE anti-mouse IgM Antibody (BioLegend UK LTD, catalog #406508); IgA Monoclonal Antibody (mA-6E1), PE, eBioscience™ (Fisher Scientific AG, catalog #12-4204-83); PE anti-mouse IgD Antibody (BioLegend UK LTD, catalog #405706); APC anti-mouse CD19 Antibody (BioLegend UK LTD, catalog #152410); PE anti-mouse Ig light chain λ Antibody (BioLegend UK LTD, catalog #407308); 4C2 (PDB: 5DO2; generated in-house); FY1 (generated in-house); MGH2 (generated in-house); Goat anti-Mouse IgG (H+L) Poly-HRP Secondary Antibody (ThermoFisher; catalog #32230)
Validation	MARV4, MARV7, MARV11, MARV12, MARV14, MARV16, MARV18, MARV20, MARV21, MARV23, MR78, MR191, EBOV-515, 4C2, FY1, and MGH2 binding to the target antigen was validated with multiple binding assays. The binding epitope of MARV16 was further identified cryoEM.<br><br>The reactivity of the remaining antibodies were validated by the manufacturer. Validation information is available on the manufacturer's website.

Eukaryotic cell lines

Policy information about cell lines and Sex and Gender in Research

Cell line source(s)	HEK-293T, VeroE6, and BS-C-1 cells were obtained from ATCC. Expi293 cells were obtained from Thermo Fisher Scientific. BHK-21/WI-2 cells were obtained from Kerafast.
Authentication	None of the cell line used were authenticated.
Mycoplasma contamination	Cells lines were not tested for mycoplasma contamination.
Commonly misidentified lines (See ICLAC register)	No commonly misidentified cells lines were used in this study.

Animals and other research organisms

Policy information about studies involving animals; ARRIVE guidelines recommended for reporting animal research, and Sex and Gender in Research

Laboratory animals	For the antibody discovery study, ATX-GK and ATX-GL female mice, 6-7 weeks old, were obtained from Alloy Therapeutics Inc.<br><br>For the immunogenicity study, 6-8 -week-old BALB/C mice (Mus musculus) were obtained from Inotiv.<br><br>For the challenge study, 4-8 week old Hartley Guinea Pigs (Cavia porcellus) were obtained from Charles River Laboratories.
Wild animals	This study did not include wild animals.
Reporting on sex	Only female mice were used for the antibody discovery study.<br><br>For the immunogenicity study, 30 male and 30 female mice were used. Each group contained 5 male and 5 female mice.<br><br>Only female Hartley Guinea Pigs were used for the challenge study.
Field-collected samples	This study did not involve field-collected samples.
Ethics oversight	For the antibody discovery work, animal experiments were performed in accordance with the Swiss Federal Veterinary Office guidelines and authorized by the Cantonal Veterinary (approval no. 35554 TI-39/2023/2023).<br><br>For the immunogenicity study, experiments were performed under the BIOQUAL Institutional Animal Care and Use Committee (IACUC)-approved Protocol no. 23-054P.<br><br>For the challenge study, experiments were approved by Texas Biomed's IACUC under protocol #1915C prior to the initiation of the study and performed in accordance with the Animal Welfare Act and the Guide for the Care and Use of Laboratory Animals.

Note that full information on the approval of the study protocol must also be provided in the manuscript.

Plants

Seed stocks	N/A
Novel plant genotypes	N/A
Authentication	N/A

Flow Cytometry

Plots

Confirm that:

☒ The axis labels state the marker and fluorochrome used (e.g. CD4-FITC).

☒ The axis scales are clearly visible. Include numbers along axes only for bottom left plot of group (a 'group' is an analysis of identical markers).

☒ All plots are contour plots with outliers or pseudocolor plots.

☒ A numerical value for number of cells or percentage (with statistics) is provided.

Methodology

Sample preparation	As described in the method section,the mice were sacrificed and peripheral blood, spleen and lymph nodes (LN) were collected and cells freshly isolated. B cellsfrom either freshly isolated or frozen splenocytes were enriched by positive selection using mouse CD19 microbeads and LS columns (Miltenyi) and subsequently stained with mouse anti-IgM, anti-IgD, anti-IgA and biotinylated MARV GPΔMuc labeled with both streptavidin- Alexa-Fluor 488 and streptavidin-Alexa-Fluor 647 (Life Technologies). MARV GPΔMuc -specific IgG+ memory B cells were sorted by flow cytometry via gating out IgM/IgD/IgA-positive B cells and positively baiting B cells with dual-labeled (Alexa-Fluor 488 and Alexa-Fluor 647) antigen, using SH800SFP cell sorter (Sony).
Instrument	Samples were acquired on FACS Symphony A1 (BD)
Software	Flow cytometry data was generated using FACS DIVA (Version 9.0). The flow cytometry data were analysed using FlowJo (version 10.8.0).
Cell population abundance	From Spleen and LNs of ATX mice, we sorted a total of 22'756 memory B cells specific for MARV GPΔMuc (See gating strategy)
Gating strategy	Gating strategies for sorting of memory B cells specific for MARV GPΔMuc are in the Figure 2b.

☒ Tick this box to confirm that a figure exemplifying the gating strategy is provided in the Supplementary Information.
